# Influenza A Virus Causes Histopathological Changes and Impairment in Functional Activity of Blood Vessels in Different Vascular Beds

**DOI:** 10.3390/v14020396

**Published:** 2022-02-15

**Authors:** Vladimir Marchenko, Irina Zelinskaya, Yana Toropova, Tatyana Shmakova, Ekaterina Podyacheva, Dmitry Lioznov, Irina N. Zhilinskaya

**Affiliations:** 1 Smorodintsev Research Institute of Influenza, Russian Ministry of Health, 197376 St. Petersburg, Russia; dlioznov@yandex.ru (D.L.); irina@influenza.spb.ru (I.N.Z.); 2 Almazov National Medical Research Centre, Russian Ministry of Health, 197341 St. Petersburg, Russia; irina.selinskaja@gmail.com (I.Z.); yana.toropova@mail.ru (Y.T.); tanesta12@gmail.com (T.S.); katrinstanford@gmail.com (E.P.)

**Keywords:** Wistar rats, influenza virus A(H1N1)pdm09, blood vessels, lungs, mesentery, histology, immunohistochemistry, wire myography

## Abstract

It has been established that blood vessels are a target for influenza virus; however, the mechanism by which virus affects the cardiovascular system remains unknown. The aim of the study is the identification of histological changes and changes in the functional activity of the pulmonary and mesenteric blood vessels of Wistar rats. Wistar rats were intranasally infected with the influenza A(H1N1)pdm09 virus. At 24 and 96 h post infection (hpi), histopathological changes were observed in lung tissues with the absence of histological changes in mesenteric tissues. The functional activity of pulmonary and mesenteric arteries was determined using wire myography. In pulmonary arteries, there was a tendency towards an increase in integral response to the vasodilator and a decrease in the integral response to the vasoconstrictor at 24 hpi (compared with control). At 96 hpi, a tendency towards a decrease in the integral response to the vasoconstrictor persisted, while the response to acetylcholine was slightly increased. The functional activity of the mesenteric blood vessels was inverted: a significant decrease in the integral response to the vasodilator and an increase in the response to the vasoconstrictor at 24 hpi were observed; at 96 hpi, the integral response to the vasoconstrictor persisted, while the response to the vasodilator remained significantly reduced. Obtained data indicate the development of endothelial dysfunction in non-lethal and clinically non-severe experimental influenza virus infection.

## 1. Introduction

Influenza virus selectively infects and damages the epithelium of the respiratory tract but can also infect other tissues and organs, including the nervous and cardiovascular system [1,2]. The first data indicating the possibility of cardiovascular disorders in patients with influenza were published by clinicians back in the 1950s [3,4,5]. In the influenza pandemics of 1918–1920 and 2009 and epidemics in 2015–2016, particularly severe clinical manifestations and consequences were noted, including skin and mucous membranes hemorrhages, nosebleeds, microhematuria, acute respiratory distress syndrome (ARDS), disseminated intravascular coagulation syndrome (DIC), hemorrhagic pulmonary, and cerebral edema [6,7,8,9]. Hemorrhagic syndrome usually occurs in severe and fulminant forms of influenza [10].

As shown by epidemiological and clinical studies, a high incidence of cardiovascular pathology in influenza is noted, especially in patients with comorbidities, including any chronic respiratory condition and cardiovascular disorders [11,12]. There is an opinion that the 1918 pandemic (Spanish flu) led to the subsequent widespread incidence of cardiovascular pathology in the first half of the 20th century [13,14]. Subsequently, it was shown that these disorders are mainly associated with damage to the vascular endothelium in different organs and tissues that have receptors (sialic acids with an α2, 6 glycosidic bond), which are necessary for virus adsorption [15,16,17]. It has now been established that the vascular endothelium and hemostatic system are targets for the influenza virus [16,18,19,20]. However, this aspect of pathogenesis is currently not taken into consideration in the development of treatment approaches for influenza, and the mechanism by which the virus affects the cardiovascular system remains unknown.

To study this mechanism, we developed an experimental model of non-lethal influenza virus infection in mature Wistar rats [21]. These laboratory animals are also one of the main experimental models for the study of various cardiovascular disorders [22,23,24]. The study was carried out for the first time on both pulmonary and mesenteric blood vessels in order to analyze local and possible systemic effects on the cardiovascular system in non-lethal and clinically non-severe experimental influenza virus infection.

## 2. Materials and Methods

### 2.1. Animals

Thirty male Wistar rats (8 to 10 weeks old) weighing 220–250 g were obtained (Preclinical Translational Research Centre, Saint-Petersburg, Russia) and divided into three groups, including two experimental groups and one control group (*n* = 10). The rats were housed in shoe-cages with two per cage. Animals were maintained at 22 ± 2 °C and a relative humidity of 50 ± 10% with a 12 h light/dark cycle. All rats received water and food ad libitum. All procedures were approved by the Animal Care and Use Committee of Research Institute of Influenza and were carried out in accordance with the principles of humane treatment of animals, regulated by the requirements of the European Convention (Strasbourg, 1986).

### 2.2. Virus

Influenza A/St. Petersburg/48/16 H1N1(pdm09) virus was obtained from the Laboratory of Influenza Evolutionary Variability of the Smorodintsev Research Institute of Influenza (St. Petersburg, Russia). The isolate selection was based on clinical studies of IAV H1N1(pdm09), as a causative agent of hemorrhagic pneumonia [25]. The virus was preliminarily adapted through 9 successive passages through infected lung homogenates of Wistar rats, as described [21]. At the last passage, the infectious activity of influenza virus was 6–7 lg EID_50_/mL and the hemagglutination titer of the virus was 1:1024.

### 2.3. Experimental Influenza Virus Infection

Rats from each experimental group after anesthesia with isoflurane (Abbott, Chicago, IL, USA) were intranasally inoculated with 0.2 mL of adapted influenza virus. Rats in the control group were anesthetized, instilled intranasally with 0.2 mL of alpha-MEM, and necropsied at 24 and 96 h. The body weight of rats was recorded before virus or alpha-MEM administration, as well as before necropsy.

### 2.4. Tissue Lavage and Homogenization of Collected Tissues

After necropsy, lungs and mesentery were aseptically removed and placed in a pre-weighed petri dish; then, the weights of the lungs and mesentery were determined. The superior lobe of the right lung and ventral part of the upper segments of the left lung were used to isolate pulmonary arteries for further functional activity determination. The remaining parts of the lungs were used for histological and immunohistochemistry assays.

Then, the lung tissues were placed in a centrifuge tube (Techno Plastic Products AG, Trasadinge, Switzerland) and the alpha-MEM (BioloT, St. Petersburg, Russia) was added (ratio of 1:10). The tissues were homogenized with the laboratory homogenizer SHM2 (Stuart, Staffordshire, England, UK) and 10% lung homogenate was centrifuged for 15 min at 1000× *g*. The mesenteric tissues after the isolation of three arteries were placed in a centrifuge tube, and then 1 mL of alpha-MEM (BioloT, St. Petersburg, Russia) was added, followed by tissue homogenization (same conditions). The supernatant was collected, then stored and aliquoted at −70 °C until the determination of the virus infectious titer.

### 2.5. Determination of Influenza Virus Infectivity Titer in Embryonated Chicken Eggs

Viral titer was determined for lung and mesenteric supernatants using influenza virus propagation in 10–12-day-old embryonated chicken eggs and expressed as lg EID_50_/mL [26]. Briefly, 10-fold serial dilutions of the supernatant (10^−1^–10^−8^) were prepared in 4.5 mL of PBS (0.01 M phosphate-buffered saline, pH 7.2), and then 0.2 mL from each dilution was used to infect embryonated chicken eggs. After the incubation (36 °C for 48 h), 100 μL of allantoic fluid was taken separately from each embryonated egg and placed in a 96-well plate (Medpolymer, St. Petersburg, Russia), with the following addition of 100 μL of 0.5% chicken erythrocytes. Viral infectious activity was calculated using the Reed and Muench method [26].

### 2.6. Histological Analysis

Middle, inferior, and post-caval lobes of the right lung and middle and lower region of the left lung and mesenteric tissues were fixed in 10% neutral buffered formalin for 24–48 h at room temperature. Histological processing was performed by the automatic tissue processor Tissue-Tek VIP 5 (Sakura, Los Angeles, CA, USA). From paraffin embedded tissue block sections with a thickness of 3 μm were made on a rotary microtome Accu-Cut SRM 200 (Sakura, Los Angeles, CA, USA). Sections were dewaxed in xylene, dehydrated in alcohols, and stained with hematoxylin and eosin. Photomicrographs were obtained using a Nikon Eclipse E400 microscope (Nikon, Minato-ku, Tokyo, Japan).

### 2.7. Immunohistochemistry

Sections of paraffin embedded tissues blocks with a thickness of 4–5 μm were placed on poly-L-lysine glass slides (Thermo Fisher Scientific, Waltham, MA, USA,). Endogenous peroxidase was inactivated with 3% H_2_O_2_ for 10 min and washed in 2 changes of Tris PBS (TBS). The blocking of non-specific staining was achieved with 1% bovine serum albumin for 20 min. The blocking serum was drained and replaced with primary murine monoclonal antibody (anti-influenza virus A antibody that recognize nucleoprotein) obtained at the Biotechnology Department of the Smorodintsev Research Institute of Influenza, (Clone 6D11) at a dilution of 1:500 for 1 h in a humid chamber. After washing twice in TBS, secondary anti-murine goat antibody conjugated with horseradish peroxidase in a 1:1000 dilution was applied for 30 min at room temperature. To detect NP expression, the visualization system Envision Flex was used, which included DAB chromogen (Dako, Glostrup, Denmark) applied for 2–3 min. Then, sections were washed three times, counterstained with Mayer’s hematoxylin, dehydrated in isopropyl alcohol, and coverslipped.

### 2.8. Determination of Functional Activity of Blood Vessels

The vascular function of pulmonary and mesenteric arteries was studied using a wire myograph DMT 620M (Danish Myo Technology, Hinnerup, Midtjylland, Denmark). Immediately after the necropsy of the rat, the heart, lungs, and mesentery were removed and placed in a Petri dish filled with a cooled Krebs–Henseleit solution of the following composition (mM): 119 NaCl, 4.7 KCl, 1.17 KH_2_PO_4_, 1.6 CaCl_2_, 1.2 MgSO_4_, 25 NaHCO_3_, 5.5 glucose, 0.03 EDTA, pH 7.4. Further manipulations to isolate the pulmonary and mesenteric arteries were performed using a binocular loupe, placing the Petri dish that was affixed to an ice pack. From the upper lobe of the right lung, sections of the lateral arteries of the 2–3rd order were isolated. From the ventral part of the upper segments of the left lung, 2nd order arteries were isolated. Three 3rd order arteries were isolated from the mesentery.

The blood vessels were mounted in the myograph chamber using two wires with a diameter of 40 μm. Three vessels from each animal in a group were studied (*n* = 30). After the normalization of the transmural pressure, the vasocontraction was activated by incubation in Krebs–Henseleit solution with increased potassium ((mM): 78.2 NaCl, 60 KCl, 1.17 KH_2_PO_4_, 1.6 CaCl_2_, 1.2 MgSO_4_, 25 NaHCO_3_, 5.5 glucose, 0.03 EDTA) and 10 μM serotonin (5-TH), followed by repeated washing with Krebs–Henseleit solution. To study the vasocontraction, the protocol of cumulative dose-dependent response to serotonin was used. The vessel was incubated in solutions with a serotonin concentration from 10^−7^ to 10^−5^ M. To study the endothelium-dependent relaxation, the vessel was preliminarily contracted with serotonin by 60% of the maximum. Then, incubation with acetylcholine (aCh) was performed according to a scheme similar to the contractile response. The data were recorded using the LabChart 8 software. For the dose-dependent curves obtained, the concentration providing 50% of the maximum response (EC50, μM), the magnitude of the response at the maximum agonist concentration (Emax, %), as well as the area under the curve were calculated.

To study the vasocontraction of the mesenteric arteries, phenylephrine (PE) was used instead of serotonin using the same procedure described above.

### 2.9. Statistical Analysis

Statistical data processing was performed using Student’s parametric test, two-way analysis of variance (ANOVA), and nonlinear regression using MS Office Excel 2016 and GraphPad Prism 8. Differences were considered statistically significant for *p* values < 0.05. Descriptive statistics such as standard deviation or standard error of the mean were used to present the obtained data.

## 3. Results

### 3.1. Body Weights; Clinical Observations

Body weights of rats were within the normal range, and no statistically significant differences between the groups was observed. No signs of clinical illness were observed in infected rats over time.

### 3.2. Influenza Virus Infectivity Titer in Pulmonary and Mesenteric Tissues

At 24 h post infection, the viral infectivity titer in the pulmonary tissues of infected rats was 6.5 lg EID_50_/mL and at 96 hpi it was significantly decreased—2.2 lg EID_50_/mL (Table 1). In the mesentery tissues, there was no virus infectivity titer over time. In control rats’ pulmonary and mesenteric tissues, a virus infectivity titer was also not registered over time.

### 3.3. Histological Assay of Pulmonary and Mesentery Blood Vessels

**Figure 1:**
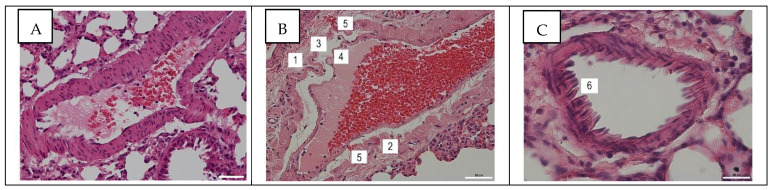
Histological examination of pulmonary tissues and blood vessels of rats in the control group (**A**) and at 24 (**B**) and 96 h post infection (**C**) with influenza A/St. Petersburg/48/16 (H1N1)pdm09 virus. Magnification ×200 (**A**,**B**); ×400 (**C**); H&E staining. (1) Stratification of adventitia; (2) stratification of media into separate muscle bundles; (3) thinning of the media; (4) thinning of the endothelium; (5) foci of erythrocyte extravasation; (6) endothelium in the form of stockade.

Various histopathological changes were found in most of the pulmonary blood vessels throughout the study. In the arteriole, the following pathological changes were observed at 24 h post infection (Figure 1B) in comparison with the control (Figure 1A): stratification of the vascular wall, media detachment from the endothelium and adventitia, stratification of media and adventitia in places, the thinning of the endothelial layer with desquamation, and small foci of erythrocyte extravasation. In arteriole at 96 hpi, a pronounced alteration in the endothelium morphology in the form of a stockade was observed (Figure 1C).

**Figure 2:**
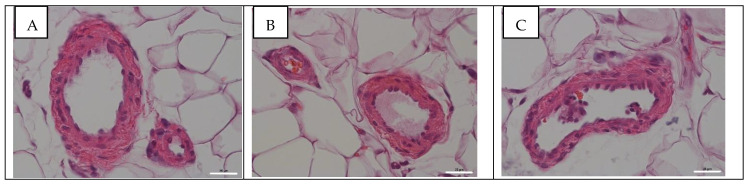
Histological examination of tissues and blood vessels of the lungs and mesentery of rats in the control group (**A**) and 24 (**B**) and 96 h after infection (**C**) with influenza A/St. Petersburg/48/16 (H1N1)pdm09 virus. Magnification ×400; H&E staining.

In mesenteric blood vessels, no histopathological changes at 24 hpi (Figure 2B) and 96 hpi (Figure 2C) were observed in comparison with the control (Figure 2A).

### 3.4. Detection of Influenza A Virus NP Antigen in Pulmonary and Mesenteric Tissues and Blood Vessels

**Figure 3:**
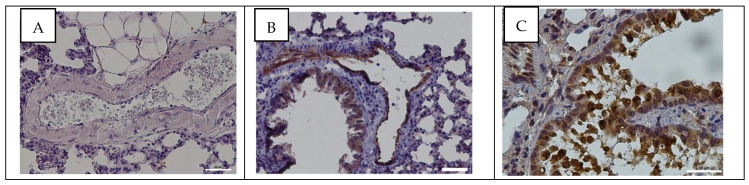
Immunohistochemical assay of pulmonary tissues and blood vessels of rats in the control group (**A**) and at 24 (**B**) and 96 h post infection (**C**) with influenza A/St. Petersburg/48/16 (H1N1)pdm09 virus using anti-NP Mabs (clone 6D11). Magnification ×200; DAB chromogen staining.

To confirm the reproduction of the virus in pulmonary and mesenteric tissues and the blood vessels of the rats, the NP antigen was detected using immunohistochemistry assay. The viral antigen was confined to rats infected with adapted influenza virus. At 24 h post infection, NP antigen was present in the endothelium of the pulmonary arteriole. NP antigen was also found in the ciliated epithelium of the bronchiole and in the alveoli (Figure 3B). At 96 hpi, NP antigen was also detected in the arteriole endothelium. In addition, NP antigen was found in the ciliated epithelium of the bronchioles (Figure 3C). Immunohistochemistry did not detect NP antigen of influenza A virus in sections of blood vessels and pulmonary tissues of rats in the control group (Figure 3A).

**Figure 4:**
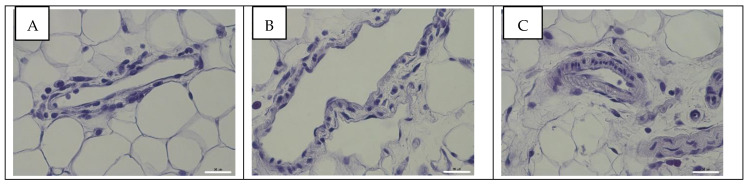
Immunohistochemical assay of mesenteric tissues and blood vessels of rats in the control group (**A**) and at 24 (**B**) and 96 h post infection (**C**) with influenza A/St. Petersburg/48/16 (H1N1)pdm09 virus using anti-NP Mabs (clone 6D11). Magnification ×400; DAB chromogen staining.

Influenza A virus NP antigen was not detected in mesentery tissues and blood vessels of the rats in the control and experimental groups (Figure 4).

### 3.5. Functional Activity of Pulmonary and Mesenteric Arteries

**Figure 5:**
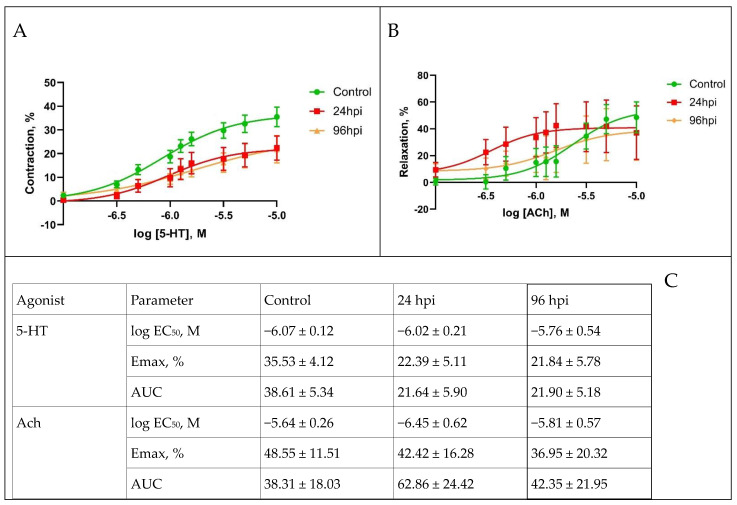
Functional activity of pulmonary arteries in the control group and at 24 and 96 h post infection with influenza A/St. Petersburg/48/16 (H1N1)pdm09 virus. (**A**): Dose-dependent response curves to serotonin (5-HT). (**B**): dose–response curves of concentration response to acetylcholine (Ach). (**C**): Maximum response of pulmonary arteries to serotonin and acetylcholine. Values represent mean ± standard error mean from 30 arteries of 10 rats in every group. * *p* < 0.05 versus control group (Dunnett’s test).

The functional activity of pulmonary arteries of rats infected with adapted influenza virus was modulated throughout the entire study (Figure 5). The analysis of dose-dependent curves revealed the following: at 24 hpi, there was a tendency of attenuation in the maximal response to a vasoconstrictor (serotonin) in pulmonary arteries and an increase in the maximal response to a vasodilator (acetylcholine) at different concentrations (Figure 5A). At 96 hpi, attenuation in maximal response to serotonin persisted on the same level, while the response to acetylcholine varied depending on the concentration of the agonist (Figure 5B). At the same time, the sensitivity of the pulmonary arteries to these agonists did not change.

The maximal response to vasoconstrictor (serotonin, 5-HT) in pulmonary arteries at 24 hpi compared with the control (35.53%) was attenuated at 22.39%. At 96 hpi, the maximal response to serotonin in pulmonary arteries was 21.84%. In turn, the maximal response to vasodilator (Ach) at 24 hpi compared with the control (48.55%) was slightly decreased to 42.42% and at 96 hpi constituted 36.95% (Figure 5C).

**Figure 6:**
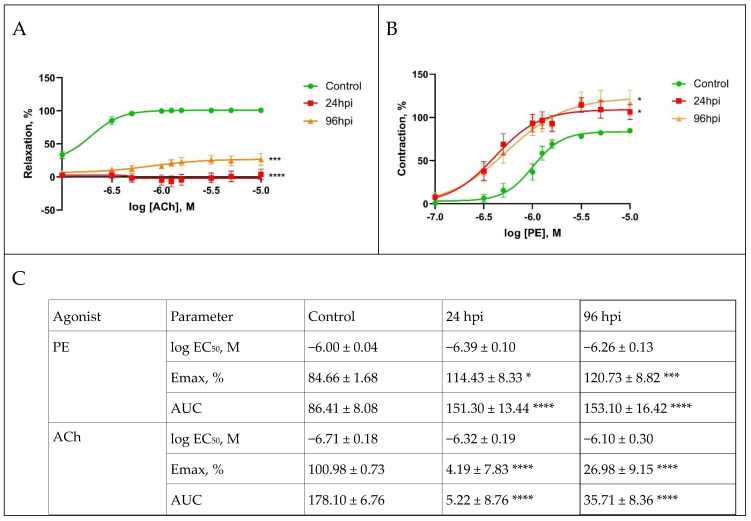
Functional activity of mesenteric arteries in the control group and at 24 and 96 h post infection with influenza A/St. Petersburg/48/16 (H1N1)pdm09 virus. (**A**): Dose-dependent response curves to phenylephrine (PE). (**B**): dose–response curves to acetylcholine (ACh). (**C**): Maximum response of pulmonary arteries to serotonin and acetylcholine. Values represent mean ± standard error mean from 30 arteries of 10 rats in every group. * *p* < 0.05, *** *p* < 0.001, **** *p* < 0.0001 versus control group (Dunnett’s test).

The functional activity of mesenteric arteries was modulated throughout the entire study (Figure 6). The analysis of dose-dependent curves showed the following changes: at 24 hpi, the maximal response to the vasoconstrictor (phenylephrine) of the mesenteric arteries was increased, while the response to the vasodilator (acetylcholine) was significantly attenuated (Figure 6A). At 96 hpi, the increase in the maximal response to phenylephrine remained increased, while the response to acetylcholine was also significantly attenuated, although slightly lesser than at 24 h post infection (Figure 6B).

Maximal response of the mesenteric arteries to phenylephrine at 24 hpi compared with the control (84.65%) was increased to 114.43%, and at 96 hpi it was 120.72% (Figure 6C). The maximal response of the mesenteric arteries to acetylcholine at 24 hpi compared with the control (100.98%) was significantly attenuated to 4.19%. At 96 hpi, the maximal response to acetylcholine was also attenuated and constituted 26.95%.

## 4. Discussion

In the present study, the experimental influenza virus infection of Wistar rats was conducted for two reasons: (1) the possibility of studying non-lethal influenza infection [21]; (2) rats of this stock are the main animal model of various cardiovascular disorders [22,23,24], which is an essential factor for our further studies.

In this study, we found that adapted influenza A/St. Petersburg/48/16 (H1N1)pdm09 virus actively reproduces in pulmonary tissues. Thus, at 24 hpi, the viral infectivity titer in pulmonary tissues was 6.6 ± 0.2 lg EID_50_/mL. At 96 hpi, the infectivity titer of influenza virus was significantly decreased to 2.2 ± 0.3 lg EID_50_/mL. No infectious influenza virus was found in mesentery. The body weight of infected rats remained unchanged throughout the entire study, and there were no clinical symptoms of the disease, which is consistent with the data obtained earlier in Sprague-Dawley and Fischer-344 rats [27,28]. The reproduction of influenza virus was confirmed by the detection of NP antigen of the influenza A virus in pulmonary tissues and blood vessels. At the same time, the expression of NP antigen in the endothelium of pulmonary blood vessels was detected throughout the study (at 24 and 96 hpi).

The histological analysis of pulmonary blood vessels at 24 hpi showed various histopathological changes. In a number of large and medium-sized vessels, the endothelium was disintegrated from the media, and the media was disintegrated from the adventitia. There was a partial stratification of the media and adventitia. In some places, there was a thinning of endothelial cells, desquamation, as well as extravasation of erythrocytes. Small-caliber blood vessels and capillaries were found with moderate spasm and desquamation of the endothelium, while the alteration of the endothelium morphology in the form of a stockade was observed. The disintegration of the endothelium from the media was less common in the large vessels at 96 hpi. Endothelial desquamation was not observed, extravasation was absent. However, despite a significant decrease in the viral infectivity titer in pulmonary tissues at 96 hpi, the endothelium of the stockade type was found more often than at 24 hpi. Histopathological changes in mesenteric blood vessels were not observed.

The functional activity of the pulmonary blood vessels of rats infected with the influenza virus was modified throughout the entire study. At 24 h post infection, there was a tendency to a decrease in maximal response to the vasoconstrictor (serotonin) and an increase in the maximal response to the vasodilator (acetylcholine). At 96 hpi, a tendency in decreasing in the maximal response to serotonin persisted, while the maximal response to acetylcholine was slightly increased. In turn, changes in the functional activity of mesenteric blood vessels were more pronounced. So, at 24 hpi, the maximal response to the vasoconstrictor (phenylephrine) was increased, and the response to the vasodilator (acetylcholine) was significantly reduced. At 96 hpi, the maximal response to phenylephrine persisted, while the response to acetylcholine remained significantly reduced. Thus, the response of pulmonary arteries and mesenteric arteries to vasoconstriction and vasodilator was utterly different, which reflects the physiology of these tissues.

All the above indicates that influenza A/St. Petersburg/48/16 (H1N1)pdm09 virus causes significant changes in blood vessels at 24 hpi in Wistar rats. At 96 hpi, a significant decrease in viral infectivity titer in pulmonary tissues was observed, which, however, was not correlated with histopathological changes in the pulmonary blood vessels, which persist and may progress. In addition, the NP antigen of the influenza virus was detected in pulmonary blood vessels at 24 hpi as well as at 96 hpi.

It is noteworthy that the impaired functional activity of mesenteric arteries in infected rats occurred in the absence of the reproduction of the influenza virus directly in the mesenteric tissues.

Probably, this can be explained by the fact that such significant changes observed in pulmonary blood vessels during influenza virus infection can serve as a trigger for the impairment of functional activity in mesenteric blood vessels and indicate a systemic effect of influenza A virus on blood vessels in influenza virus infection. It is a fact that the vascular endothelium is the main regulator of hemostasis; therefore, impairment in the functional activity of pulmonary blood vessels in influenza virus infection leads to the dysregulation of the hemostasis system, which includes coagulation, fibrinolysis, platelet aggregation, etc. It is likely that this dysregulation can affect the functioning of the vascular wall of other blood vessels in different tissues and organs. All these data point to the necessity of expanding influenza therapy including medications with endothelial and vaso-protective effect. However, it is difficult to say which medications should be administered. Some research groups used statins for influenza treatment [29]. However, efficacy of statins in patients with flu is debatable [30,31]. It is possible that statins do not eliminate endothelial dysfunction in influenza. Obviously, we need to have more information about the mechanisms of endothelial dysfunction in influenza virus infection, which will help us to select effective medications with endothelial and vaso-protective effect. The screening of such medications will be the aim of our future research.

Obtained results indicate that non-lethal and clinically non-severe experimental influenza virus infection causes histopathological changes and impairment in the functional activity of blood vessels in different vascular beds, which may be the cause of severe disease course. In addition, our result can serve as a basis for expanding the influenza therapy and including medications with endothelial and vaso-protective effect in treatment regimens.

## Figures and Tables

**Table 1:** Infectious titer of influenza A/St. Petersburg/48/16 H1N1(pdm09) virus in pulmonary and mesentery tissues of infected rats. Standard deviations for 5 repeats are shown (*: *p* < 0.05).

Hours Post Infection, hpi	Virus Titer (lg EID_50_/mL)	
	In Lungs	In Mesentery
24	6.5 ± 0.2 *	0.0
96	2.2 ± 0.3 *	0.0

## Data Availability

Not applicable.
